# Laparoscopic versus Open Surgery in Lateral Lymph Node Dissection for Advanced Rectal Cancer: A Meta-Analysis

**DOI:** 10.1155/2019/7689082

**Published:** 2019-03-05

**Authors:** Manzhao Ouyang, Tianyou Liao, Yan Lu, Leilei Deng, Zhentao Luo, Jinhao Wu, Yongle Ju, Xueqing Yao

**Affiliations:** ^1^Department of Gastrointestinal Surgery, Shunde Hospital, Southern Medical University, Shunde, Foshan, Guangdong Province 528300, China; ^2^Department of General Surgery, Guangdong General Hospital (Guangdong Academy of Medical Sciences), Guangzhou, Guangdong Province 510080, China; ^3^The Second School of Clinical Medicine, Southern Medical University, Guangzhou, Guangdong Province 510080, China; ^4^China-American Cancer Research Institute, Dongguan Scientific Research Center, Guangdong Medical University, Dongguan, Guangdong Province 523808, China

## Abstract

**Aim:**

To compare the clinical efficacies between laparoscopic and conventional open surgery in lateral lymph node dissection (LLND) for advanced rectal cancer.

**Methods:**

We comprehensively searched PubMed, Embase, Cochrane Library, CNKI, and Wanfang Data and performed a cumulative meta-analysis. According to inclusion criteria and exclusion criteria, all eligible randomized controlled trials (RCTs) or retrospective or prospective comparative studies assessing the two techniques were included, and then a meta-analysis was performed by using RevMan 5.3 software to assess the difference in clinical and oncological outcomes between the two treatment approaches.

**Results:**

Eight studies involving a total of 892 patients were finally selected, with 394 cases in the laparoscopic surgery group and 498 cases in the traditional open surgery group. Compared with the traditional open group, the laparoscopic group had a longer operative time (WMD = 81.56, 95% CI (2.09, 142.03), *P* = 0.008), but less intraoperative blood loss (WMD = −452.18, 95% CI (-652.23, -252.13), *P* < 0.00001), shorter postoperative hospital stay (WMD = −5.30, 95% CI (-8.42, -2.18), *P* = 0.0009), and higher R0 resection rate (OR = 2.17, 95% CI (1.14, 4.15), *P* = 0.02). There was no significant difference in the incidence of surgical complications between the two groups (OR = 0.52, 95% CI (0.26, 1.07), *P* = 0.08). Lateral lymph node harvest, lateral lymph node metastasis, local recurrence, 3-year overall survival, and 3-year disease-free survival did not differ significantly between the two approaches (*P* > 0.05).

**Conclusion:**

Laparoscopic LLND has a similar efficacy in oncological outcomes and postoperative complications to the conventional open surgery, with the advantages of reduced intraoperative blood loss, shorter postoperative hospital stay, and higher R0 resection rate, and tumor radical cure is similar to traditional open surgery. Laparoscopic LLND is a safe and feasible surgical approach, and it may be used as a standard procedure in LLND for advanced rectal cancer.

## 1. Introduction

Compared with colon cancer, patients with rectal cancer often undergo local recurrence after radical surgery, which not only affects the prognosis but also seriously threatens the quality of life of patients. A growing evidence has suggested that lateral lymph node (LLN) metastasis is a major cause of local recurrence of advanced rectal cancer [1]. According to several multicenter studies in Japan, the incidence of lateral lymph node metastasis (LLNM) in low rectal cancer is 13.3-20.1% [2, 3]. However, in the treatment strategy of LLNM, whether or not to perform lateral lymph node dissection (LLND) is the main controversy between Western countries and Asian countries headed by Japan. Especially in advanced low rectal cancer, neoadjuvant chemoradiotherapy combined with total mesorectal excision (TME) is the standard treatment in Europe and the United States, but Japanese guidelines for rectal cancer recommend TME combined with LLND treatment [4].

At present, LLND usually still adopts open surgical methods, but based on the results of several RCTs, laparoscopic surgery, as a minimally invasive surgery, has been widely accepted in colon cancer surgery with oncological outcomes comparable to those of open surgery [5, 6]. However, in rectal surgery, especially for low rectal cancer, TME is a complex procedure in the narrow pelvis, and furthermore, TME combined with LLND requires longer operative time and leads to more blood loss than TME alone [7]. Therefore, whether laparoscopic LLND can be used as an alternative surgical method for LLND is still controversial, although some short-term superior outcomes have been reported in some studies of laparoscopic LLND versus open LLND, such as less blood loss, reduced complication rates, and shorter recovery periods [8–10]. However, most of these reports are defective, such as a majority of retrospective studies, small sample size, a lack of long-term observations, and contradictory results between the different studies. Therefore, laparoscopic LLND has not been well resolved in these studies, and it remains to be determined whether laparoscopic LLND is safe and feasible in clinical and oncological outcomes.

In view of this, we conducted a meta-analysis, based on the published literature on laparoscopic versus open LLND in the past, to evaluate the feasibility and oncologic safety of laparoscopic LLND, further to provide reference for clinicians to choose surgical approvals in the future.

## 2. Materials and Methods

This meta-analysis was prepared in accordance with the recommendations from the Preferred Reporting Items for Systematic Reviews and Meta-Analyses (PRISMA) statement [11].

### 2.1. Literature Search Strategy

PubMed, Cochrane Library, Embase, CNKI, and Wanfang Data searches were comprehensively done on all relevant studies between 01-Jan-1990 and 10-Jun-2018 that compared laparoscopic LLND with open LLND in patients with radical resection of rectal cancer. Having said that the CNKI and Wanfang Data are Chinese databases, the following MeSH terms and their combinations were searched in [Title/Abstract]: *laparoscopic*, *open*, “*rectal neoplasm*^∗^”, “*rectum neoplasm*^∗^”, “*rectal tumor*^∗^”, “*cancer of rectum*”, “*rectal cancer*^∗^”, “*rectum cancer*^∗^”, “*cancer of the rectum*”, “*lateral lymph node dissection*”, “*lateral lymph node*”, “*lateral pelvic lymph node dissection*”, “*lateral pelvic lymph node*”, “*lateral pelvic wall lymph-node dissection*”, “*extended lymphadenectomy*”. The related-articles function was used to broaden the search, and the computer search was supplemented with manual searches of the reference lists of all retrieved studies, review articles, and conference abstracts. All relevant articles identified were assessed, and preestablished inclusion and exclusion criteria were applied.

### 2.2. Inclusion Criteria

The following are the inclusion criteria of the study: (1) The above databases were used from 01-Jan-1990 to 10-Jun-2018 to search published literature in English or Chinese. (2) The literature included retrospective studies, prospective RCTs, and well-designed non-RCTs. (3) Literatures have a similar purpose, design, and statistical methods. (4) Interventions were done for laparoscopic LLND versus open LLND. (5) Pooled results can be formulated by the statistical index, such as odds ratio (OR), weighted mean difference (WMD), relative risk (RR), or hazard ratio (HR).

### 2.3. Exclusion Criteria

The following are the exclusion criteria for the study: (1) The sample size is too small and the number of cases is less than 20 cases. (2) The articles could not acquire original data or full text, or the articles were not written in Chinese or English. (3) The literatures were published by the same researcher or research institutes, the most recent or most informative one was included, but the research that subjects or observed results of studies were different could be selected. (4) The count data or measurement data of the original literature follow-up truncation were not clear or could not be obtained by calculation. (5) Other treatments were differently performed between two groups during pre- and post-operation, and these treatments probably affected the prognosis of patients.

### 2.4. Literature Screening, Data Extraction, and Outcomes of Interest

The titles and abstracts of all the literatures were carefully read and examined to exclude obviously unrelated documents. The full text of ambiguous literature was deeply read to determine whether it is included. Data extraction forms were established, and information provided in the literatures was fully extracted. All steps were independently conducted and cross-checked by three reviewers (MZOY, ZTL, and YLJ) and resolved by discussion with adjudicating senior authors (XQY) in case of disagreement. Data extraction tables include the following: first author, publication year, grouping method, sample size, literature source, study location, intervention method, operation time, intraoperative blood loss, postoperative hospital stay, surgical complication rate, local recurrence rate, 3-year overall survival rate, 3-year disease-free survival rate, and basic data of the subjects (gender, age, etc.). The lack of information is supplemented by contacting the original author by telephone or e-mail.

### 2.5. Quality Assessment

We used the modified Newcastle-Ottawa Quality Assessment Scale (NOS) [12] to assess the quality of the included studies. The NOS scores were based on three main factors: method of patient selection, comparability of the study groups, and assessment of outcome. Scores of 0–9 points were allocated to each study. Studies achieving six or more points were considered to be of high quality. Quality assessment was performed independently by three authors (MZOY, ZTL, and YLJ). In case discrepancies arose, articles were reexamined and consensus was reached by discussion; the same method has been used for data extraction.

### 2.6. Statistical Processing

Meta-analysis was performed by using RevMan 5.3 software provided by Cochrane Collaboration. The statistics of the count data were expressed by OR and 95% CI, and the statistics of the measurement data were expressed by WMD and 95% CI. Assessment of statistical heterogeneity between the studies was undertaken using the *χ*^2^ and *I*^2^ statistical tests. There was no statistical heterogeneity between the studies when the *P* value was ≥0.1 or *I*^2^ ≤ 50%, and the fixed effect model was used for meta-analysis. Conversely, there was statistical heterogeneity between the studies when *P* value < 0.1 or *I*^2^ > 50%, and a random effects model was used for meta-analysis. Publication bias assessment was performed through Begg's test and funnel plot representation by using STATA SE version 12.0. A two-tailed *P* value of less than 0.05 was considered statistically significant. Sensitivity analyses of this study were performed for high-quality studies [13–18]. For the literature providing median and range continuity variables, the standard deviation (SD) and variance were extracted using the method described by Hozo et al. [19]. The extraction of log(HR) and SE was carried out by using the method described by Tierney et al. [20]. These continuity variables that only provided quartiles and mean and standard deviation which cannot be extracted were eliminated.

### 2.7. Subgroup Analysis

Considering some differences between laparoscopic surgery and robotic-assisted laparoscopic surgery in LLND, subgroup meta-analyses were performed based on a surgical approach between laparoscopic surgery and robotic-assisted laparoscopic surgery to explore the potential heterogeneity and avoid the interference and influence caused by the two different surgical methods.

## 3. Results

### 3.1. Study Selection

**Figure 1:**
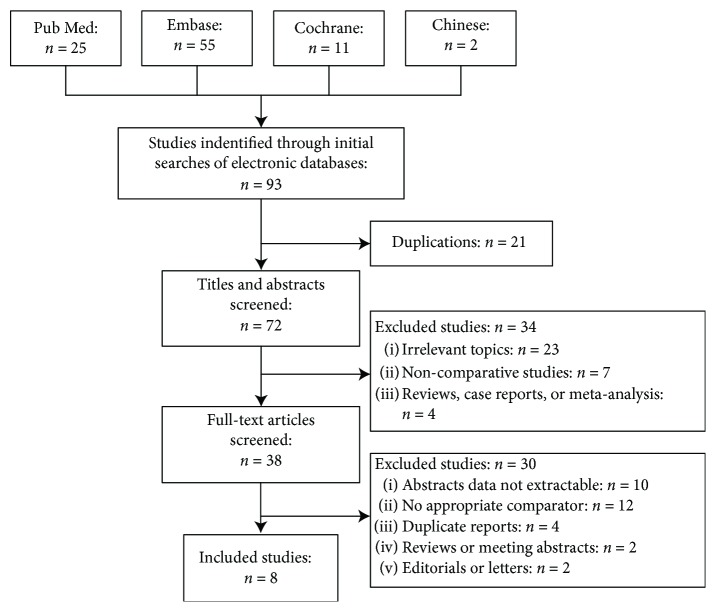
Flow diagram of study selection.

The study selection process is shown in the flow-process diagram (Figure 1). 93 publications related to the initial inspection and 21 duplicate publications were eliminated by using EndNote X8 software combined with manual checking. 34 were excluded after skimming through titles and abstracts. The remaining 38 studies were fully read. Of these, 30 publications were excluded with all kinds of reasons as shown in Figure 1. Finally, eight studies [13–18, 21, 22] were included in the final analysis. Examination of the references listed for these studies and for the review articles did not yield any further studies for evaluation.

### 3.2. Characteristics and Methodological Quality of Eligible Studies

Eight studies including 892 cases (394 cases for laparoscopic LLND and 498 cases for open LLND) fulfilled the predefined inclusion and exclusion criteria and were included in the meta-analysis. Three studies had a possible overlapping population, but were included since they investigated different outcomes, and one of these studies is a multicenter study. There were two prospective non-RCTs and six retrospective studies. The study characteristics, patient demographic details, selection criteria, matching, and quality scoring for each study are shown in Table 1.

### 3.3. Meta-Analysis Results

#### 3.3.1. Operation Time

**Figure 2:**
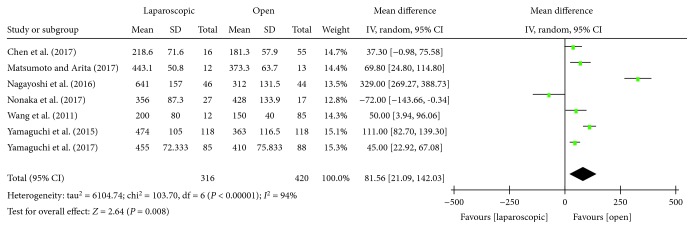
Forest plot and meta-analysis of intraoperative operative time.

There were seven studies [14–18, 21, 22] that reported the operative time of laparoscopic and open LLND. Statistical heterogeneity was present in this study (*P* < 0.00001, *I*^2^ = 94%) using a random effects model. Meta-analysis showed that the operation time of the laparoscopic group was longer than that of the open group, and the difference was statistically significant [WMD = 81.56, 95% CI (2.09, 142.03), *P* = 0.008] (Figure 2).

#### 3.3.2. Estimated Blood Loss

**Figure 3:**
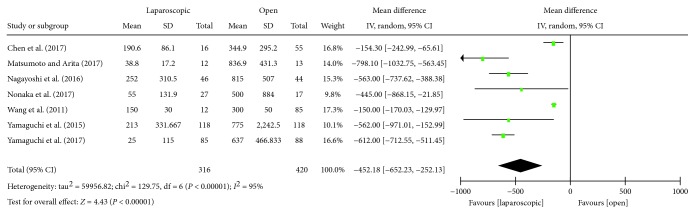
Forest plot and meta-analysis of intraoperative blood loss.

There were seven studies [14–18, 21, 22] that compared the volume of intraoperative blood loss in laparoscopic and open LLND. There was statistical heterogeneity in the study (*P* < 0.00001, *I*^2^ = 95%), using a random effects model. Meta-analysis showed that the amount of blood loss in the laparoscopic group was lower than that in the open group, and the difference was statistically significant [WMD = −452.18, 95% CI (-652.23, -252.13), *P* < 0.00001] (Figure 3).

#### 3.3.3. Postoperative Hospital Stay

**Figure 4:**
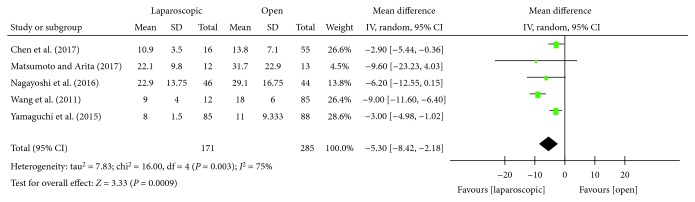
Forest plot and meta-analysis of postoperative hospital stay.

The length of postoperative hospital stay was reported in five studies [16–18, 21, 22]. Statistical heterogeneity was found to be high (*P* = 0.003, *I*^2^ = 75%), hence the random effects model was used. Meta-analysis showed that the length of postoperative hospital stay in the laparoscopic group after LLND was significantly shorter than that in the open group, and the difference was statistically significant [WMD = −5.30, 95% CI (-8.42, -2.18), *P* = 0.0009] (Figure 4).

#### 3.3.4. Postoperative Complications

**Figure 5:**
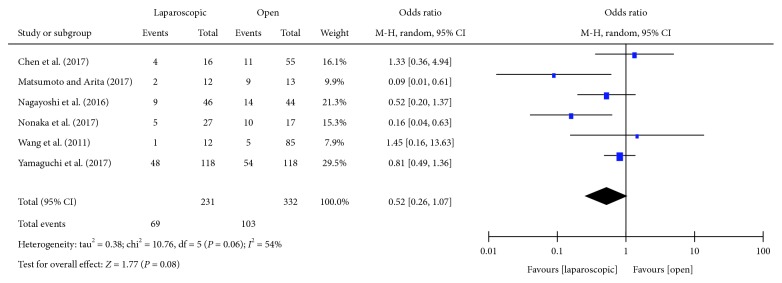
Forest plot and meta-analysis of postoperative complications.

The incidence of postoperative complications was reported in six studies [14–16, 18, 21, 22]. There was significant heterogeneity between the studies (*P* = 0.06, *I*^2^ = 54%); thus, the random effects model was used. There was no statistical significant difference between the two groups in postoperative complications [OR = 0.52, 95% CI (0.26, 1.07), *P* = 0.08] (Figure 5).

#### 3.3.5. Lateral Lymph Node Harvest

**Figure 6:**
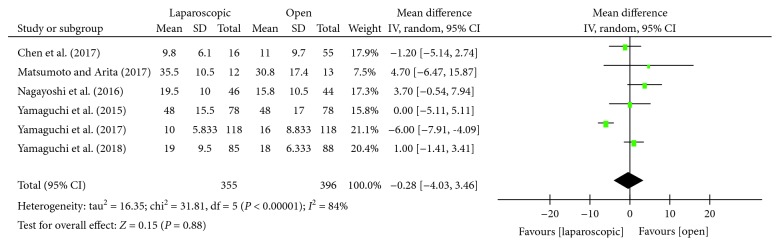
Forest plot and meta-analysis of the number of lateral lymph node harvest.

Six studies [13, 15–18, 22] assessed the number of lateral lymph node harvests among laparoscopic and open LLND. Statistical heterogeneity was found to be high (*P* < 0.00001, *I*^2^ = 84%); hence, the random effects model was used. Meta-analysis showed no statistically significant difference in the number of lateral lymph nodes between the laparoscopic group and the open group [WMD = −0.28, 95% CI (-4.03, 3.46), *P* = 0.88] (Figure 6).

#### 3.3.6. Lateral Lymph Node Metastasis

**Figure 7:**
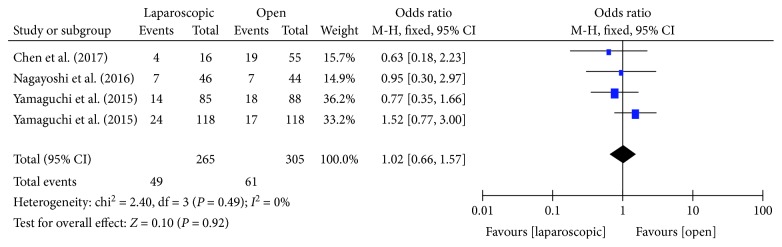
Forest plot and meta-analysis of lateral lymph node metastasis.

There were four studies [15, 17, 18, 22] that compared the positive lymph nodes in the lateral lymph nodes obtained in the laparoscopic and open LLND. There was no heterogeneity between the studies (*P* = 0.49, *I*^2^ = 0%); thus, the fixed effects model was used. Meta-analysis showed no statistically significant difference in the rate of lateral lymph node metastasis between the two groups [OR = 1.02, 95% CI (0.66, 1.57), *P* = 0.92] (Figure 7).

#### 3.3.7. R0 Resection Status

**Figure 8:**
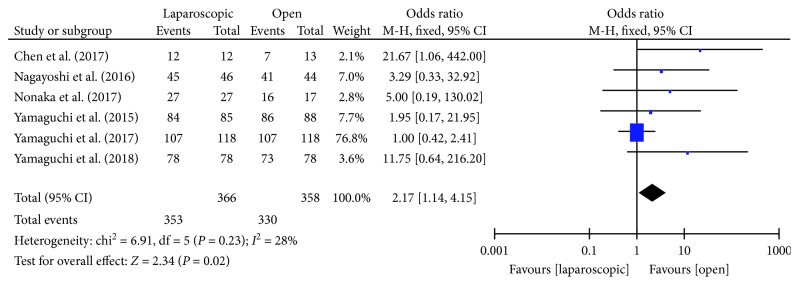
Forest plot and meta-analysis of R0 resection rate.

Six studies [13–18] reported the R0 resection status of laparoscopic and open surgery in LLND. There was moderate heterogeneity between the studies (*P* = 0.23, *I*^2^ = 28%); thus, the fixed effects model was used. Meta-analysis showed that laparoscopic LLND had a higher R0 resection rate than in the open group [OR = 2.17, 95% CI (1.14, 4.15), *P* = 0.02] (Figure 8).

#### 3.3.8. Local Recurrence Rate

**Figure 9:**
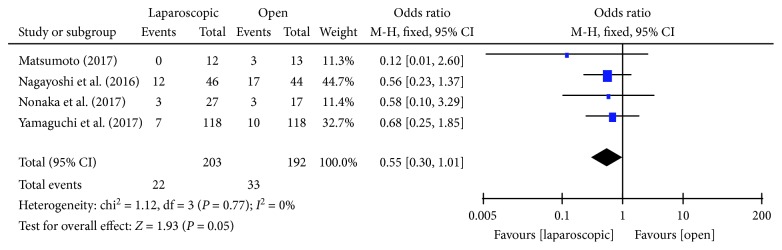
Forest plot and meta-analysis of local recurrence rate.

Four studies [14–16, 18] reported the local recurrence rate after laparoscopic or open LLND. Statistical homogeneity was not found in each study (*P* = 0.77, *I*^2^ = 0%); hence, the fixed effects model was used. There was no significant difference in the local recurrence rate of LLND between the laparoscopic and open techniques [OR = 0.55, 95% CI (0.30, 1.01), *P* = 0.05] (Figure 9).

#### 3.3.9. 3-Year Overall Survival

**Figure 10:**
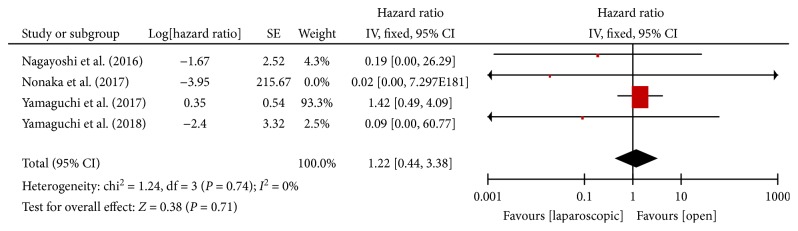
Forest plot and meta-analysis of 3-year OS.

There were four studies [13–15, 18] that reported the 3-year overall survival rates after laparoscopic or open LLND. There was no heterogeneity between the studies (*P* = 0.74, *I*^2^ = 0%); thus, the fixed effects model was used. Meta-analysis showed no significant difference in 3-year overall survival between laparoscopic and open LLND [HR = 1.22, 95% CI (0.44, 3.38), *P* = 0.71] (Figure 10).

#### 3.3.10. 3-Year Disease-Free Survival

**Figure 11:**
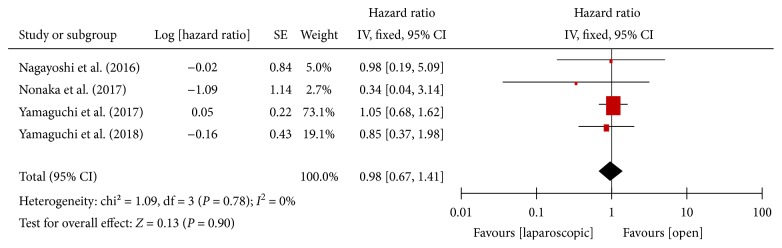
Forest plot and meta-analysis of 3-years DFS.

No heterogeneity was found between the studies [13–15, 18] for the 3-year disease-free survival (*P* = 0.78, *I*^2^ = 0%). Meta-analysis showed no statistically significant difference in 3-year disease-free survival between the laparoscopic and open approaches [HR = 0.98, 95% CI (0.67, 1.41), *P* = 0.90] (Figure 11).

#### 3.3.11. Publication Bias Analysis and Sensitivity Analysis

**Figure 12:**
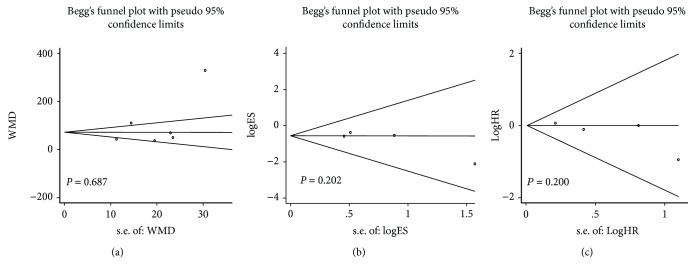
Begg's funnel diagram: (a) operative time; (b) local recurrence rate; (c) 3-year DFS.

Figure 12 shows the funnel plots included in the study for operative time, local recurrence rate, and 3-year DFS. No significant publication bias was observed in Begg's test (*P* > 0.05).

Except for two Chinese studies, six Japanese studies including two prospective studies and four retrospective studies [13–18] that scored six or more points on the modified NOS were included in the sensitivity analysis (Table 2). There was no change in the significance of any of the outcomes except for postoperative complications, which was shown to be significantly lower in the laparoscopic group than in the open group (OR = 8.04, 95% CI (0.15, 0.91), *P* = 0.03). The degree of between-study heterogeneity decreased slightly for estimated blood loss and postoperative hospital stay but not for other observed results.

#### 3.3.12. Subgroup Analysis

The results of subgroup meta-analysis (Table 3) showed no significant differences between laparoscopic surgery and robotic-assisted laparoscopic surgery in operative time, estimated blood loss, postoperative hospital stay, lateral lymph node harvest, lateral lymph node metastasis, 3-year OS, and 3-year DFS. However, after subgroup analysis in terms of R0 resection status, there were no significant differences in both laparoscopic LLND vs open LLND and robotic-assisted laparoscopic LLND vs open LLND, respectively. In addition, subgroup differences among all outcomes between laparoscopic LLND vs open LLND and robotic-assisted laparoscopic LLND vs open LLND were not significant (*P* > 0.05).

## 4. Discussion

### 4.1. Rectal Cancer and Lateral Lymph Node Dissection

In the radical resection of rectal cancer, there has been much controversy about whether or not to conventionally perform lateral lymph node dissection. However, there are increasing evidences that LLND can benefit patients. According to Japanese reports, even though bilateral lymph node was defined negatively by computed tomography (CT) scanning or magnetic resonance imaging (MRI) for the patients of low rectal cancer, 7.4% of patients in the LLND group were found to have LLNM [23], while the patients in whom TME + LLND was performed had a local recurrence rate reduced by about 50% and the 5-year survival rate of patients with rectal cancer increased by 8-9% [7, 23, 24]. In particular, a recent multicenter randomized controlled trial in Japan showed that patients with preoperative stages II and III and no lateral lymph node metastasis failed to demonstrate the noninferiority of TME surgery compared with TME plus LLND. TME + LLND can reduce the local recurrence rate, especially the recurrence of the lateral wall [25].

### 4.2. Laparoscopy and Lateral Lymph Node Dissection

Compared with traditional open surgery, laparoscopic surgery has been widely used in rectal cancer surgery because of its low intraoperative blood loss, light pain, rapid postoperative recovery, and no obvious influence on appearance. Some scholars believe that laparoscopy with high-resolution magnified images and illumination systems may have certain advantages in the treatment of LLND in patients with rectal cancer in the deep and narrow pelvic cavity. However, considering that TME combined with LLND requires a longer operation time and more intraoperative blood loss than does single TME surgery [23], laparoscopic LLND is technically challenging and difficult; thus, the most surgical approaches of LLND in Japan are still open. Therefore, there is still no consensus on the choice of surgical approaches for LLND. At present, there are few studies on the clinical efficacy of laparoscopic versus open surgery in LLND for rectal cancer. Some of the existing studies are mostly retrospective uncontrolled studies, small sample size, a lack of long-term observations, and a certain discrepancy between different studies. Therefore, whether laparoscopic LLND can be used as an alternative surgical approach remains doubtful and requires a higher level of evidence to demonstrate.

### 4.3. Perioperative Outcomes

In this study, we analyzed three perioperative results of two surgical approaches including operative time, intraoperative blood loss, and postoperative hospital stay. Although the operative time of the laparoscopic group was longer than that of the open group, the laparoscopic group had less intraoperative blood loss and shorter postoperative hospital stay than the open group. These findings might be attributed to a magnified and clear surgical field offered by laparoscopy for surgeons in the narrow and deep pelvic cavity, which facilitates accurate anatomy and secure hemostasis. In addition, some of the advantages of robotic-assisted laparoscopic lateral lymph node dissection (RALLD) include free-moving multiple joint forceps, high-quality three-dimensional imaging, and stable camera operation, which help doctors perform a more detailed anatomy and achieve better visual effects in the narrow pelvis. Laparoscopic LLND has a longer time than traditional open surgery and has high requirements for the surgeon. However, we believe that the surgeons' surgical skills of laparoscopic LLND will be improved and operative time will be shortened through strict training.

### 4.4. Postoperative Complications

Postoperative complications of LLND mainly include wound infection, postoperative bleeding, intestinal obstruction, anastomotic leakage, urinary dysfunction, urinary tract infection, and sexual dysfunction. Dysuria and sexual dysfunction are still the major complications of affecting patients' quality of life after rectal cancer resection with LLND. Hojo et al. [26] reported that 40%-100% of patients had sexual dysfunction after enlarged lymph node dissection of rectal cancer, and the incidence of urinary dysfunction reached 7%-70% [27, 28] caused by lateral lymph node dissection. To reduce high postoperative complications, Hojo et al. [27] proposed LLND with autonomic nerve preservation in 1991. The complete preservation of the autonomic nerve can reduce the incidence of urinary dysfunction by 4%-8%, which can significantly improve the quality of life of postoperative patients. With the accumulation of surgical experience, similar results can be achieved in patients with non-LLND. Saito et al. [29] found that the incidence of urogenital dysfunction between the TME + LLND group and TME group were 79% and 68%, respectively, by following 343 male patients with rectal cancer, with no statistical difference, and TME + LLND have not increased the incidence of genitourinary dysfunction.

There was no significant difference in the rate of grade 3/4 complications (Clavien-Dindo Classification) between the laparoscopic and open groups [30]. According to Yamaguchi et al. [17], the incidence of wound infection, small bowel obstruction, and anastomotic leakage was lower in the RALLD group compared with the open LLND group. In this study, the total incidence of postoperative complications was not statistically significant (*P* = 0.08). However, according to the results of sensitivity analysis, the postoperative complications were significantly lower in the laparoscopic group than in the open group in Table 2. On the one hand, the difference might be attributed to the heterogeneity of included studies. On the other hand, the discrepancy might be associated with the fact that we only compared total complication between two groups instead of separately analyzing each complication for two groups. Some scholars believe that [14] in the perspective of urinary dysfunction, laparoscopic LLND is superior to open LLND in accurate anatomy and better visualization through a larger surgical field of view, which is beneficial to protecting autonomic nerves. In addition, due to the low incidence of postoperative complications, postoperative hospital stay was significantly shorter in the laparoscopic group.

### 4.5. Oncologic Outcomes and Prognosis

The pathological parameters of evaluating the quality of rectal surgery and oncology results included the number of LLN harvest, the rate of LLNM, the R0 resection rate, the local recurrence rate, the 3-year OS rate, and the 3-year DFS rate. In our study, the R0 resection rate of laparoscopic surgery was higher than that of open surgery, although there was no significant difference between the laparoscopic group and the open group in the number of harvested LLN and the rate of LLNM. Some scholars considered that the technical advantages of laparoscopic surgery for safe pelvic dissection might be beneficial for the rate of safe resection margins or resecting a sufficient number of lymph nodes [17]. The long-term survival rate and tumor recurrence rate are the accepted criteria for judging whether the surgery follows the principle of tumor-free technique. Our study showed that there was no difference in local recurrence, 3-year OS, and 3-year RFS between the two groups for rectal cancer. Therefore, laparoscopic LLND can be considered to achieve the same clinical efficacy as open surgery. Although some randomized controlled trials of TME have demonstrated that laparoscopic surgery was no worse than open surgery [31, 32], there are few long-term prognostic reports about TME combined with LLND. Nagayoshi et al. [18] reported that there was no significant difference between the 3-year OS and the 3-year DFS between the laparoscopic LLND and open LLND groups, consistent with our results. Therefore, laparoscopic LLND can be considered to achieve the same long-term efficacy as open surgery. In addition, further research on the long-term prognosis of laparoscopic LLND is necessary in the future because of lack of high-quality RCTs.

In this study, taking into consideration the difference between laparoscopic surgery and robotic-assisted laparoscopic surgery and the outcomes between the two surgical methods might be different. Therefore, subgroup analysis was performed based on a surgical approach between laparoscopic LLND and RALLD to explore the potential heterogeneity and the potential influence to final results. From the results of the subgroup analysis (Table 3), most of the results before and after subgroup analysis did not change significantly. After the subgroup analysis, the R0 resection rates of laparoscopic LLND and RALLD did not become significant, which may be related to the smaller sample size of separate two subgroups. Therefore, the positive effect of surgical outcomes might not be dominantly influenced by robotic-assisted laparoscopic surgery.

### 4.6. Limitations

There are some limitations in this meta-analysis as the following. (1) All included studies are non-RCTs that can increase the risk of selection bias and reduce the evidence intensity of this meta-analysis. (2) There is a certain degree of clinical heterogeneity because of the differences of qualifications of the surgeons and the differences of different patients in each study, which may have a certain impact on the results. (3) The included literature is not comprehensive enough because this study only selects published literature reported in English or Chinese, which may cause a selection bias in this study. The above various reasons inevitably affect the intensity of evidence in the evaluation results of this meta-analysis.

## 5. Conclusion

This meta-analysis indicates that laparoscopic surgery is a safe and feasible surgical approval in TME combined with LLND for advanced rectal cancer, which can achieve adequate oncological safety and long-term survival comparable to that of open surgery. Furthermore, laparoscopic approval has the advantages of minimally invasive surgery such as cosmetic results, less trauma, rapid recovery, and meticulous dissection, which will promote laparoscopic LLND to become a better alternative to lateral lymph node dissection for advanced rectal cancer. However, despite our rigorous methodology, future large-scale, well-designed RCTs with extensive follow-up are expected to verify and update the findings of this analysis, because the inherent limitations of included studies prevent us from reaching definitive conclusions.

## Figures and Tables

**Table 1:** Characteristics of included studies.

Study	Year	Area	Design	Type	Patients (*n*)		TNM stage, *n*, I/II/III/IV		Neoadjuvant therapy (*n*)		Matching	Quality score
					L/RL	O	L/RL	O	L/RL	O		
Matsumoto and Arita	2017	Japan	R	L	12	13	NR	NR	NR	NR	1, 2, 3, 5, 9	7
Nagayoshi et al.	2016	Japan	R	L	46	44	NR	NR	6	26	1, 2, 3, 7, 8, 9, 10	7
Yamaguchi et al.	2018	Japan	R	RL	78	78	15/18/45/0^¶^	11/20/47/0^¶^	6	6	1, 2, 3, 4, 5, 6, 7, 8, 10	8
Yamaguchi et al.	2017	Japan	R	L	118	118	0/31/86/0*^ζ^*	0/28/90/0*^ζ^*	28	28	1, 2, 3, 4, 5, 6, 7, 8	8
Yamaguchi et al.	2015	Japan	P	RL	85	88	0/19/59/7*^ζ^*	0/22/58/8*^ζ^*	10	11	1, 2, 3, 4, 5, 6, 7, 8, 9, 10	6
Nonaka et al.	2017	Japan	P	L	27	17	7/5/15/0^¶^	2/3/12/0^¶^	5	1	1,2,7,8,10	7
Chen et al.	2017	China	R	L	16	55	3/2/11/0*^ζ^*	2/11/42/0*^ζ^*	6	9	1,2,7,9,10	5
Wang et al.	2011	China	R	L	12	85	NR	NR	NR	NR	NR	4

R = retrospective; P = prospective; L = laparoscopic surgery; RL = robotic-assisted laparoscopic surgery; O = open surgery; NR = not reported; TNM stage ¶ = pTNM stage; *ζ* = cTNM stage. Matching: 1 = age; 2 = sex; 3 = body mass index; 4 = American Society of Anesthesiologists score; 5 = tumor distance from anal verge; 6 = previous abdominal surgery; 7 = neoadjuvant chemoradiotherapy; 8 = dissection area; 9 = tumor size; 10 = histopathologic type of tumor.

**Table 2:** Sensitivity analysis comparison of laparoscopic/robotic-assisted laparoscopic LLND and open LLND.

Outcomes of interest	Studies (*n*)	L/RL (*n*)	O (*n*)	WMD/OR/HR (95% CI)	*P* value	Study heterogeneity			
						*χ* ^2^	df	*I* ^2^ (%)	*P* value
Operative time (min)	5	288	280	96.98† (12.52-181.44)	0.02	98.49	4	96	<0.00001
Estimated blood loss (ml)	5	288	280	-615.35† (-694.06 to -536.64)	<0.00001	3.37	4	0	0.50
Postoperative hospital stay (days)	3	143	145	-3.40† (-5.27 to -1.53)	0.0004	1.70	2	0	0.43
Postoperative complications	4	203	192	0.37 (0.15-0.91)	0.03	8.78	3	66	0.03
Lateral lymph node harvest (*n*)	5	339	341	0.01 (-4.53 to 4.55)	1.00	31.57	4	87	<0.00001
Lateral lymph node metastasis	3	249	250	1.09 (0.69-1.74)	0.70	1.76	2	0	0.42
R0 resection status	6	353	330	2.17 (1.14-4.15)	0.02	6.91	5	28	0.23
Local recurrence	4	203	192	0.55 (0.30-1.01)	0.05	1.12	3	0	0.77
3-year overall survival	4	269	257	1.22^∗^ (0.44-3.38)	0.71	1.24	3	0	0.74
3-year disease-free survival	4	269	257	0.98^∗^ (0.67-1.44)	0.90	1.09	3	0	0.78

L = laparoscopic surgery; RL = robotic-assisted laparoscopic surgery; O = open surgery; WMD = weighted mean difference; OR = odds ratio; HR = hazard ratio; CI = confidence interval; df = degrees of freedom. ^∗^HR; †WMD.

**Table 3:** Subgroup analysis between laparoscopic LLND and robotic-assisted laparoscopic LLND.

Outcomes of interest	Studies (*n*)	L/RL (*n*)	O (*n*)	WMD/OR/HR (95% CI)	*P* value	Subgroup difference			
						*χ* ^2^	df	*I* ^2^ (%)	*P* value
Operative time (min)									
L vs O	6	231	332	88.03† (9.20-166.87)	0.03	1.06	1	5.8	0.30
RL vs O	1	85	88	45.00† (22.92-67.08)	<0.00001				
Estimated blood loss (ml)									
L vs O	6	231	332	-407.11† (-585.92 to -228.31)	<0.00001	3.83	1	73.9	0.05
RL vs O	1	85	88	-612.00† (-712.55 to -511.45)	<0.00001				
Postoperative hospital stay (days)									
L vs O	4	113	214	-6.27† (-10.43 to -2.11)	0.003	1.94	1	48.5	0.16
RL vs O	1	85	88	-3.00† (-4.98 to -1.02)	0.003				
Lateral lymph node harvest (*n*)									
L vs O	4	192	230	-0.56† (-6.01 to 4.89)	0.84	0.21	1	0	0.65
RL vs O	2	163	166	0.82† (-1.37 to 3.00)	0.46				
Lateral lymph node metastasis									
L vs O	3	180	217	1.17 (0.69-1.97)	0.56	0.78	1	0	0.38
RL vs O	1	85	88	0.77 (0.35-1.66)	0.50				
R0 resection status									
L vs O	4	203	192	1.80 (0.89-3.67)	0.10	1.14	1	12.6	0.28
RL vs O	2	163	166	5.08 (0.88-29.40)	0.07				
3-year overall survival									
L vs O	3	191	179	1.30^∗^(0.46-3.66)	0.62	0.63	1	0	0.43
RL vs O	1	78	78	0.09^∗^(0.00-60.77)	0.47				
3-year disease-free survival									
L vs O	3	191	179	1.01^∗^(0.67-1.52)	0.97	0.12	1	0	0.73
RL vs O	1	78	78	0.85^∗^(0.37-1.98)	0.71				

L = laparoscopic surgery; RL = robotic-assisted laparoscopic surgery; O = open surgery; VS = versus; WMD = weighted mean difference; OR = odds ratio; HR = hazard ratio; CI = confidence interval; df = degrees of freedom. ^∗^HR; †WMD.

## Abbreviations

LLND: Lateral lymph node dissection
LLN: Lateral lymph node
LLNM: Lateral lymph node metastasis
RCTs: Randomized controlled trials
CNKI: China National Knowledge Infrastructure
TME: Total mesorectal excision
RALLD: Robotic-assisted laparoscopic lateral lymph node dissection
OS: Overall survival
DFS: Disease-free survival
HR: Hazard ratio
OR: Odds ratio
WMD: Weighted mean difference
SE: Standard error
CI: Confidence intervals.
